# Vascular plants from European Russia in the CSBG SB RAS Digital Herbarium

**DOI:** 10.3897/BDJ.8.e56504

**Published:** 2020-10-08

**Authors:** Nataliya Kovtonyuk, Irina Han, Evgeniya Gatilova

**Affiliations:** 1 Central Siberian Botanical Garden SB RAS, Novosibirsk, Russia Central Siberian Botanical Garden SB RAS Novosibirsk Russia

**Keywords:** collections, data paper, dataset, digital herbarium, digitisation, European Russia, GBIF, NS, NSK, ObjectScan 1600, occurrence, specimen

## Abstract

**Background:**

The Central Siberian Botanical Garden of the Siberian Branch of the Russian Academy of Sciences (CSBG SB RAS) is the largest botanical institution in the Asian part of Russia. Founded in 1946, CSBG SB RAS is historically a consortium of two herbarium collections with their own acronyms (NS and NSK) and registration in the Index Herbariorum (Thiers 2020).

At present the NS+NSK collections contain about 800,000 herbarium specimens comprising vascular plants (680,000), mosses (25,000), lichens (80,000) and fungi (15,000) gathered, not only in Siberia, but also in the European part of Russia and other parts of the Eurasian and American continents. CSBG SB RAS has the third largest collection in Russia after the Komarov Botanical Institute of RAS (LE) and Moscow State University (MW) collections.

The dataset consists of 5,384 records of digitised herbarium specimens of vascular plants belonging to 111 families, collected since the 19th century in 54 administrative regions from the European part of Russia and kept in NS+NSK collections. Herbarium specimens were digitised using two special scanners, both ObjectScan 1600, according to international standards, at 600 dpi, with a barcode, 24-colour scale and spatial scale bar and placed into the CSBG SB RAS Digital Herbarium. For each specimen, the species name, locality, collection date, collector, ecology and revision label are recorded. More than 94% of the records have coordinates that fall within the area of European Russia, west of the Ural Mountains.

**New information:**

A total of 5,384 records of vascular plant occurrences with 94.8% geolocations in the territory of the European Russia West of the Ural Mountains were entered.

## Introduction

Free and open access to biodiversity data is essential for informed decision-making to achieve conservation of biodiversity and sustainable development (Chavan and Penev 2011, Penev et al. 2017). Preserved specimen collections are the most important source of scientific information about the distribution of specimens in the past and present, which allows simulation of the dynamics of objects in the future. Only the herbarium sample reliably confirms the presence of the plant organism in a specific point of space at a certain time. Herbarium collections and the data they hold are valuable, not only for the traditional studies of taxonomy and systematics, but also for ecology, bioengineering, conservation, food security and the human social and cultural elements of scientific collection (Baird 2010, James et al. 2018). The value and universality of herbarium specimens are recognised in most countries, where national and large regional herbaria are actively developing and improving (Costello et al. 2013, Cranston et al. 2014, Kovtonyuk 2017, Pearse et al. 2017). The digitisation and open access to the collections have become a common trend in biodiversity collections management, the latest stage in improving the inventory and modernisation of herbarium collections of the leading botanical institutions in the world (Heberling et al. 2019, Le Bras et al. 2017, Seregin 2020, Kovtonyuk et al. 2019a).

With the digitisation of natural history collections over the last decades, their traditional roles for taxonomic studies and public education have been greatly expanded into the fields of biodiversity assessments, climate change impact studies, trait analyses, sequencing, 3D object analyses etc. (Nelson and Ellis 2018, Raes et al. 2019, Watanabe 2019). Herbarium specimens represents snapshots of phenological events and have been reliably used to characterise phenological responses to climate (Willis et al. 2017).

The CSBG SB RAS was founded in 1946 and currently is the largest botanical institution in the Asian part of Russia. The first herbarium collection at the CSBG SB RAS was organised in 1944 on the basis of herbarium sheets transferred from the Medical and Biological Institute (Novosibirsk), currently the collection named after I.M. Krasnoborov (NS). The NSK collection was transferred from Irkutsk in 1978, the collection named after M.G. Popov. Historically, it is a consortium of two herbarium collections with their own acronyms (NS and NSK) and registration in the Index Herbariorum.

Digitisation of vascular plants at 600 dpi was initiated in 2014 by using the herbarium scanner Herbscan (JSTOR 2020), starting with the type specimens of M.G. Popov's Herbarium (Kovtonyuk 2015). At the end of 2017, the research group "Unique scientific unit - Herbarium of higher plants, lichens and fungi (NS, NSK)" with the short name “USU-Herbarium” was organised in CSBG SB RAS for herbarium digitisation and herbarium management. The goal of the research group is to provide open access to the digitised collections of CSBG SB RAS as a worldwide data resource for the study of biodiversity. The digitisation of herbarium specimens started in 2018 using two herbarium scanners ObjectScan 1600 (Microtek 2020) according to international standards. For each specimen, the species name, locality, collection date, collector and ecology were digitised and verified (Kovtonyuk et al. 2019b). To date, more than 47,000 herbarium specimens have been digitised, verified and placed into the CSBG SB RAS Digital Herbarium (http://herb.csbg.nsc.ru:8081).

This datapaper describes the data about the herbarium specimens digitised in 2020 under the initiative "Call for data papers from European Russia", which were digitised and geolocated and the taxonomic status of the specimens was revised. The digitisation of the herbarium will be continued and the dataset will be updated in the future.

## General description

### Purpose

The purpose of this paper is to describe a dataset published in GBIF (Kovtonyuk et al. 2020) in the format of a peer-reviewed journal paper and to provide recognition for the effort by means of a scholarly article (Chavan and Penev 2011, Penev et al. 2017).

## Project description

### Title

Vascular plants from European Russia in the CSBG SB RAS Digital Herbarium

### Personnel

Nataliya Kovtonyuk, Irina Han, Evgeniya Gatilova

## Sampling methods

### Study extent

The herbarium collections of the CSGB SB RAS are mainly focused on plant collections from Siberian regions. However, both NS and NSK collections contain unique materials collected by CSBG researchers in the European part of Russia (Table 1), as well as duplicate plant sheets obtained from other herbaria (Table 2). The collection covers almost two centuries, with the oldest herbarium specimen being collected in 1831 by G.S. Karelin. Many herbarium sheets from "Herbarium Florae Ingricae" (ca. the year 1860) do not contain the exact date and place of collection. Amongst 230 specimens digitised in the CSBG SB RAS and included in the dataset, 70 specimens do not have a collection date and 160 specimens do not have an exact place of collection. Some data from the herbaria of the European part of Russia from CSBG SB RAS collections have been published previously in GBIF (GBIF.org 2020) and devoted to certain taxonomic groups. From the 5384 records included in the dataset, 5103 (94.7%) were geolocated.

### Sampling description

In both NS and NSK collections, European Russia is not separated as a single section and does not have a separate catalogue. The digitisation of the herbarium specimens fom European Russia in NS and NSK started under the "Call for data papers from European Russia". In total, 4139 herbarium specimens from the NS collection which were applicable for the target region were first digitised. Another 1245 specimens were mounted, barcoded and digitised, then accessioned in NSK and included in the collection's database. The total number of the herbarium specimens from European Russia in CSBG SB RAS ranges between 10,000 and15,000 and its digitisation will continue in the future.

Dried and pressed herbarium specimens were digitised by two special scanners ObjectScan 1600, according to international standards: at 600 dpi, with a barcode, 24-colour scale and spatial scale bar (Kovtonyuk 2015, Kovtonyuk et al. 2018a, JSTOR 2020). Images (*.jpg files) and metadata are stored in the CSBG SB RAS Digital herbarium (http://herb.csbg.nsc.ru:8081). Two integrated workstations were equipped with an ObjectScan 1600 scanner, ScanWizard_Botany software and MiVapp_Botany archive management system software (Microtek 2020) with the following parameters and modules: scan design for full-frame focus, a maximum of 1600 dpi (equal to 1 Gigabyte pixels), colour CCD, Optical Character Recognition (OCR) for specimen label and ID barcode and image archive and privileged-account cloud management system.

### Quality control

Many botanists took part in the identification of the herbarium specimens, especially Bochkin V.D., Kuzeneva O.I., Makarov V.V., Ramenskaya M.L., Orlova N.I., Reshetnikova N., Egorova T., Kovtonyuk N.K., Klochkova Z., Smirnova T., Smirnov N., Chernov E.T., Gogina E.E., Gusev Yu.D., Nepli G.N. and other specialists from the Komarov Botanical Institute of the Russian Academy of Sciences (Saint Petersburg), Tsitsin Main Botanical Garden of the Russian Academy of Sciences (Moskow), Lomonosov Moskow State University and CSBG SB RAS.

Quality control was carried out by staff of USU-Herbarium group during the verification of the digitised samples. Label metadata information was placed into Calc table (Open Office) and then modified into a table of Darwin Core Standard.

### Step description

The digitisation process included the main steps: (Kovtonyuk et al. 2018b):

1. Mounting of dry plant material on to a herbarium sheet;
2. Reviewing the identification and nomenclature by a specialist;
3. Barcoding the specimen: printing a barcode on the thermal printer and mounting it to the herbarium sheet;
4. Placing the herbarium sheet, 24-colour scale and scale bar on the scanner platform and image capturing;
5. Generating metadata, labelling OCR by ScanWizard Botany and verification of the label text by experts;
6. Archive management by MiVapp-Botany;
7. Georeferencing using Open Google map, Yandex map and other open maps.

## Geographic coverage

### Description

Herbarium specimens were collected from each of the 54 administrative regions of European Russia. The most intense collection had been done in Moscow Oblast and Moscow (866), Murmansk Oblast (833), Leningrad Oblast including Saint-Petersburg (628), Volgograd Oblast (354) and Pskov Oblast (239) (Table 3).

### Coordinates

37 and 81 Latitude; 19.6 and 65.56 Longitude.

## Taxonomic coverage

### Description

The taxonomic coverage of the dataset includes 111 families from 41 orders and 6 classes of vascular plants, following GBIF Backbone Taxonomy (GBIF Secretariat 2019). The classes represented in the dataset are: Gnetopsida (1 family), Liliopsida (21 families), Lycopodiopsida (2), Magnoliopsida (73), Pinopsida (3) and Polypodiopsida (11). The largest families in the dataset are: Poaceae (655 specimens), Cyperaceae (605), Asteraceae (504), Rosaceae (272), Fabaceae (241), Brassicaceae (224), Caryophyllaceae (207), Amaranthaceae (205), Primulaceae (187) and Lamiaceae (170).

## Traits coverage

### Data coverage of traits

PLEASE FILL IN TRAIT INFORMATION HERE

## Temporal coverage

### Notes

**Figure 1.**
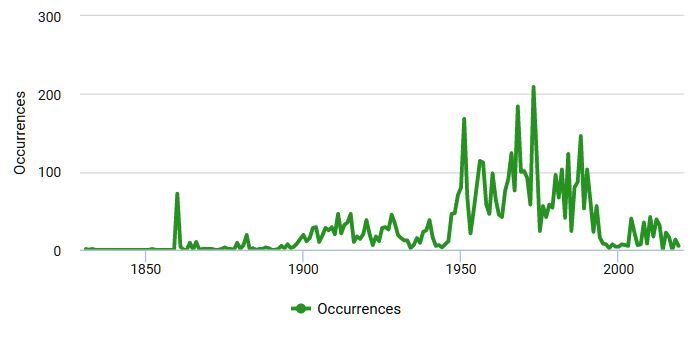
Occurrences per year

01-05-1831 through 14-08-2019 Occurrences of the vascular plants from European Russia in the CSBG SB RAS Digital Herbarium per year are shown in Figure 1.(Fig. 1)

## Collection data

### Collection name

I.M. Krasnoborov Herbarium; M.G. Popov Herbarium

### Collection identifier

NS; NSK

### Specimen preservation method

dried and pressed

## Usage rights

### Use license

Other

### IP rights notes

This work is licensed under a Creative Commons Attribution (CC-BY) 4.0 Licence.

## Data resources

### Data package title

Vascular plants from European Russia in the CSBG SB RAS Digital Herbarium

### Resource link

https://www.gbif.org/dataset/85f9137e-8aec-4e0b-9ed6-0af4dbe491e8

### Alternative identifiers

http://www.csbg.nsc.ru:8080/ipt/resource?r=euvrus

### Number of data sets

1

### Data set 1.

#### Data set name

Vascular plants from European Russia in the CSBG SB RAS Digital Herbarium

#### Data format

Darwin Core

#### Number of columns

28

#### Description

The dataset from the European part of Russia consists of 5384 records of the digitised herbarium specimens of vascular plants collected from 19th century to the present. For each specimen, the species name, locality, collection date, collector, ecology and revision label are recorded. More than 94% of the records have coordinates that fall within the area of European Russia, west of the Ural Mountains.

**Data set 1.** 

Column label	Column description
occurrenceID	An identifier for the Occurrence
CollectionCode	The acronym identifying the collection (NS or NSK)
TypeStatus	A list of nomenclatural types (type status, typified scientific name, publication) applied to the subject.
scientificName	The full scientific name, with authorship.
Genus	The full scientific name of the genus in which the taxon is classified
specificEpithet	The species epithet of the scientificName
scientificNameAuthorship	The authorship information for the scientificName formatted according to the conventions of the applicable nomenclaturalCode
infraspecificEpithet	The name of the lowest or terminal infraspecific epithet of the scientificName, excluding any rank designation
Family	The full scientific name of the family in which the taxon is classified
Order	The full scientific name of the order in which the taxon is classified
Class	The full scientific name of the class in which the taxon is classified
RecordedBy	The collector of herbarium specimen
fieldNumber	An identifier given to the event in the field
eventDate	The date-time or interval during which an Event occurred
Year	The four-digit year in which the Event occurred, according to the Common Era Calendar
Month	The ordinal month in which the Event occurred
Day	The integer day of the month on which the Event occurred
countryCode	The standard code for the country in which the Location occurs
Country	The name of the country or major administrative unit in which the Location occurs
stateProvince	The name of the next smaller administrative region than country in which the Location occurs
decimalLatitude	The geographic latitude (in decimal degrees) of the geographic centre of a Location
decimalLongitude	The geographic longitude of the geographic centre of a Location
minimumElevationInMetres	The lower limit of the range of elevation, in metres
verbatimLocality	The original textual description of the place
identifiedBy	A list of names of people who assigned the Taxon to the subject
occurrenceRemarks	Comments or notes about the Occurrence
geodeticDatum	The ellipsoid, geodetic datum, or spatial reference system (SRS) upon which the geographic coordinates given in decimalLatitude and decimalLongitude as based.
coordinateUncertaintyInMetres	The horizontal distance (in metres) from the given decimalLatitude and decimalLongitude describing the smallest circle containing the whole of the Location.

## Figures and Tables

**Table 1.** List of CSBG SB RAS collectors and regions of field trips in European part of Russia.

Collectors	Year	Regions	Number of digitised specimens
Ivanova M.M.	1962	Krasnodar Krai	9
Krasnoborov I.M.	1973	Leningrad Oblast	5
Krasnoborov I.M., Khanminchun V.N.	1974	Karachay-Cherkess Republic	14
Grankina V.P.	1983	Stavropol Krai	1
Krasnoborov I.M.	1984	Murmansk Oblast	52
Ovchinnikova S.V.	1990	Orenburg Oblast	1
Korolyuk A.Ju.	1998	Samara Oblast	5
Krasnikov A.A.	2003	Krasnodar Krai	1
Lomonosova M.N.	2004	Astrakhan Oblast Volgograd Oblast	28
Kovtonyuk N.K.	2004	Leningrad Oblast	1
Korolyuk A.Ju.	2008	Rostov Oblast	30
Lomonosova M.N.	2008	Republic of Karelia	4
Korolyuk A.Ju.	2010	Rostov Oblast	34
Kovtonyuk N.K.	2010	Stavropol Krai	3
Korolyuk E.A., Korolyuk A.Ju.	2011	Krasnodar Krai, Rostov Oblast	12
Lomonosova M.N.	2012	Volgograd Oblast, Republic of Kalmykia	37
Korolyuk A.Ju.	2015	Orenburg Oblast, Republic of Bashkortostan	14
Lashchinskiy N.N.	2015	Orenburg Oblast	6
Shaulo D.N., Doronkin V.M.	2015	Samara Oblast	2
Korolyuk A.Ju.	2016	Samara Oblast, Ulyanovsk Oblast	10
Agafonov A.V., Asbaganov S.V.	2016	Republic of Bashkortostan	6
Tomoshevich M.A., Banaev E.V.	2018	Astrakhan Oblast	11
Makryi T.V.	2018	Orenburg Oblast	2

**Table 2.** Most active collectors from European Russia in the dataset.

Collector	Specimens digitised
Kuzeneva O.I.	296
Bochkin V.D.	279
Skvortsov A.K.	266
Orlova N.I.	175
Ponomaryova L.R.	173
Chernov E.G.	168
Beljanina N.B.	137
Makarov V.V.	134
Gogina E.E.	134
Svezhenina A.F.	118
Smirnova T.	110
Korolyuk A.Ju.	105
Nepli G.N.	102
Khokhrjakov A.P.	96
Karpenko A.S.	95
Sagalaev V.A.	93
Dryahlova A.D.	91
Matsenko A.E.	76
Litvinov D.I.	75
Krasnoborov I.M.	71
Lomonosova M.N.	69
Ramenskaya M.L.	65
Rusanovich I.I.	64
Vasilevich V.I.	63
Andreev V.	61
Klinkova G.Yu.	59
Manin A.F.	56
Tikhomirov V.N.	53
Kostyleva N.V.	51
Smirnov N.	47

**Table 3.** Most presented regions from European Russia in the dataset.

Region	Specimens digitised
Moscow Oblast	865
Murmansk Oblast	833
Leningrad Oblast	628
Volgograd Oblast	354
Pskov Oblast	239
Republic of Dagestan	206
Republic of Karelia	182
Bryansk Oblast	178
Astrakhan Oblast	170
Krasnodar Krai	134
Saratov Oblast	105
Karachay-Cherkess Republic	89
Komi Republic	86
Republic of Kalmykia	85
Rostov Oblast	84
Republic of Bashkortostan	82
Stavropol Krai	72
Ryazan Oblast	67
Perm Krai	66
Kirov Oblast	65
